# Sexual Violence against Men Who Have Sex with Men and Transgender Women in Mongolia: A Mixed-Methods Study of Scope and Consequences

**DOI:** 10.1371/journal.pone.0139320

**Published:** 2015-10-02

**Authors:** Sarah M. Peitzmeier, Faiza Yasin, Rob Stephenson, Andrea L. Wirtz, Altanchimeg Delegchoimbol, Myagmardorj Dorjgotov, Stefan Baral

**Affiliations:** 1 Johns Hopkins Bloomberg School of Public Health, Baltimore, Maryland, United States of America; 2 Boston University School of Medicine, Boston, Massachusetts, United States of America; 3 University of Michigan School of Nursing, Ann Arbor, Michigan, United States of America; 4 Johns Hopkins School of Medicine, Baltimore, Maryland, United States of America; 5 UNAIDS Mongolia, Ulaanbaatar, Mongolia; 6 Youth For Health NGO, Ulaanbaatar, Mongolia; The University of New South Wales, AUSTRALIA

## Abstract

The role of sexual violence in health and human rights-related outcomes, including HIV, is receiving increasing attention globally, yet the prevalence, patterns, and correlates of sexual violence have been little-studied among men who have sex with men (MSM) and transgender women in low and middle income countries. A mixed-methods study with quantitative and qualitative phases was conducted among MSM and transgender women in Ulaanbaatar, Mongolia. Methods included respondent-driven sampling (RDS) with structured socio-behavioral surveys (N = 313) as well as qualitative methods including 30 in-depth interviews and 2 focus group discussions. Forced sex in the last three years was reported by 14.7% of respondents (RDS-weighted estimate, 95%CI: 9.4–20.1; crude estimate 16.1%, 49/307) in the quantitative phase. A descriptive typology of common scenarios was constructed based on the specific incidents of sexual violence shared by respondents in the qualitative phase (37 incidents across 28 interviews and 2 focus groups). Eight major types of sexual violence were identified, most frequent of which were bias-motivated street violence and alcohol-involved party-related violence. Many vulnerabilities to and consequences of sexual violence described during the qualitative phase were also independently associated with forced sex, including alcohol use at least once per week (AOR = 3.39, 95% CI:1.69–6.81), and having received payment for sex (AOR = 2.77, 95% CI:1.14–6.75). Building on the promising strategies used in other settings to prevent and respond to sexual violence, similar strengthening of legal and social sector responses may provide much needed support to survivors and prevent future sexual violence.

## Introduction

More than three decades into the HIV epidemic, an increasing body of research is elucidating the social and structural barriers such as sexual violence [1, 2] that fuel a disproportionate burden of HIV among key populations [3–5]. Men who have sex with men (MSM) and transgender women in low- and middle-income countries have more than 19 times higher odds of living with HIV compared to other reproductive age adults [3], while transgender women globally when studied alone had nearly 50 times higher odds of HIV infection [5]. In Mongolia, a lower-middle income country where homosexuality is legal but stigmatized and not protected by anti-discrimination legislation [6], 61% of all cumulative HIV cases observed until 2014 have been among MSM and transgender women, and HIV prevalence appears to be growing in this population [7, 8].

There has been a growth of interest in violence experienced by key populations with the increasing recognition that sexual violence is a human rights violation of public health significance [9]. Sexual and gender minorities face many types of sexual violence throughout the life course, including childhood sexual abuse, sexual intimate partner violence, and sexually violent hate crimes [10–13]. Lifetime sexual violence has been significantly associated with HIV-related risk practices among MSM, including selling sex, buying sex, drug use, history of imprisonment or jail, higher numbers of male partners, and unprotected anal intercourse [14–16]. HIV transmission may be increased for MSM and transgender women during sexual violence due to high transmission efficiency and trauma associated with forced unprotected anal receptive intercourse [17, 18]. Legal and social marginalization of sexual and gender minorities fuels both HIV[2] and violence. To develop evidence-based sexual violence-prevention interventions for MSM and transgender women, there is a need to understand factors associated with vulnerability to sexual violence and perpetrator-victim typologies.

There is relatively limited qualitative research describing the phenomenon of sexual violence against MSM and transgender women globally, with the bulk of the existing literature focused on intimate partner violence again gay and bisexual men in high-income settings. Qualitative work among Maori and other New Zealand MSM found that alcohol use played a key role in rendering men vulnerable to sexual violence. Sexual inexperience, youth, and “novelty” in the community also set up a power dynamic in which young MSM were coerced into sex by older men in the community [14, 19]. Participants in this study also noted “gay social venues” such as “parties, bars, events, and saunas” as being high-risk for sexual violence. In a sample of white British male rape victims who were predominantly gay or bisexual, perpetrators perceived to be a mix of gay-identified and straight-identified men and were usually known to the victim. Unlike the New Zealand study, most sexual violence was accomplished via physical force and most incidents took place in the perpetrator’s or victim’s home [20]. Qualitative work with American transgender adolescents describes sexual violence from male and female perpetrators, with perpetrators frequently insulting their gender identity during the assault [21]. Advocacy organizations have described the role that homophobia and transphobia play in marginalizing victims of sexual and intimate partner violence [22]. There is a need to gain a more holistic picture of the many different forms of sexual violence that MSM and transgender women experience over the life course in diverse settings. The present study aims to address this gap in the Mongolian setting.

Prevalence data on sexual violence among MSM are predominantly from the US, UK, and New Zealand and vary widely, and data for transgender women are even less conclusive. The percentage of gay and bisexual men reporting history of forced or unwanted sex ranges from 12% to 54% [10], with a recent meta-analysis estimating that 32% of gay men experience sexual violence [23]. A recent US review concluded that the “most common” estimate of lifetime sexual violence victimization is “about 50%” among transgender populations [24]. Data on sexual violence experienced by sexual and gender minorities in low and middle income countries are emergent. In Karnataka State, India 17.5% of MSM and transgender women reported past-year sexual violence and in Thailand 18.4% of MSM and transgender women reported lifetime sexual violence [16, 25]. In a nine-city study in Guatemala, El Salvador, Nicaragua, Costa Rica, and Panama, 5.1% of MSM and transgender women reported sexual violence in the past 12 months [1]. Estimates of sexual violence prevalence specific to transgender women outside the context of sex work in low and middle income countries are lacking.

This mixed-methods study presents the estimated prevalence and correlates of recent sexual violence against MSM and transgender women in Mongolia, and contextualizes sexual violence vulnerability by developing a typology describing the most common sexually violent scenarios. Although MSM and transgender women have differing sexual orientation, gender identity, gender presentation, and sexual behavior, both groups are included as there is significant overlap in these communities in Mongolia, and both groups are at significant risk for HIV and violence. Public discourse around LGBT issues has only recently begun to flourish in Mongolia, with the first LGBT rights advocacy organization registered in 2009 and the first Pride Week held in 2013 [6]. Mongolian LGBT identities are still emerging, in particular transgender identities, and MSM and transgender women have highly overlapping social networks and risk contexts for HIV and violence. However, sexual and gender minority individuals with more feminine gender expression are particularly targeted for violence and harassment in public places and employment discrimination [26]. Understanding the scope and context of sexual violence in these populations is critical for designing effective sexual violence and HIV prevention interventions.

## Methods

This convergent parallel mixed-methods study consisted of a quantitative phase (respondent-driven sampling survey) and qualitative phase (in-depth interviews and focus group discussions). Qualitative and quantitative phases occurred simultaneously in Ulaanbaatar, Mongolia between January and April, 2011. Eligibility criteria included being assigned male sex at birth, being 16 years of age or older (legal age of consent for sexual activity in Mongolia), reporting anal sex with a man within the past 12 months, and providing informed consent in Mongolian. Those who were eligible had the option to participate in one or both phases of the study. Study participants were compensated 18,000 Tugrik (US $10) for their time and transportation. The rationale of a mixed design was to provide complementarity or context, such that the qualitative data would provide a contextual understanding of the phenomena of interest while the quantitative data would provide more generalizable understanding of prevalence and allow us to test relationships between variables in the data [27].

### Quantitative Phase

Methods for the quantitative phase of this study have been described in detail elsewhere [28]. Respondent-driven sampling (RDS) is a peer-based chain recruitment method often used to reach hidden populations where enumerating a sampling frame is infeasible [29]. Following consent and enrollment, interviewers administered a structured questionnaire that took 30–45 minutes to complete in Mongolian. Participants were given three recruitment coupons and were paid 8,000 Tugrik (US $5) for each respondent recruited.

The primary analytic goal was to assess correlates of sexual violence. Sexual violence was assessed narrowly in the quantitative phase with the question “In the past three years, have you ever been forced to have unwanted sex or have you ever been raped?” A three-year time window was chosen after formative research indicated this was a meaningful reference period. Survey measures also included participant demographics; sexual behaviors; substance use; experiences of stigma and discrimination; history of imprisonment and drug/alcohol treatment; and HIV and STI diagnosis history. “Transgender” is used as an umbrella term in this analysis to describe participants who did not identify as a man. A total of 313 participants were enrolled, including 29 transgender women.

RDSAT Version 6.0.1 (Ithaca, NY), which incorporates the RDS I estimator, was used to obtain population-weighted estimates for history of sexual violence and selected risk factors [29, 30]. Results are presented in tables as crude and RDS-weighted with 95% confidence intervals (95% CI); results in text are RDS-weighted unless otherwise indicated. Bivariate logistic regression analyses assessing the relationship between recent sexual violence and selected characteristics were calculated using STATA v11.2/IC (StataCorp, College Station, TX) statistical software. Listwise deletion was used to handle missing data. A conceptual framework of sexual violence risk factors and sequelae was developed with concepts deriving from the qualitative data and informed the selection of potential covariates of recent forced sex among survey items. Items that were statistically significant (p<0.05) in bivariate analyses were included in a multivariate logistic regression model. Backward elimination with a *p* value set to 0.1 was used to determine the final multivariate model. Variables that were significantly (p<0.05) associated with sexual violence in the multivariate model were reported by presenting adjusted odds ratios (AOR), 95% confidence intervals and *p* values. RDS weights were not used to construct the regression models, as unadjusted estimates for advanced modeling are considered more conservative statistically [31].

### Qualitative Phase

Qualitative study participants were accrued via purposive sampling from the wider MSM and transgender female population in Ulaanbaatar as well as from respondents from the quantitative phase. Thirty in-depth interviews (IDIs) were conducted: MSM living with HIV (n = 11), HIV-uninfected MSM (n = 12), and transgender women (n = 7). Each IDI was conducted with a unique participant and lasted an average of 70 minutes. Two focus group discussions (FGDs) were conducted: one FGD with three transgender women and one FGD with five MSM. Each FGD lasted approximately 2.75 hours. Interviews and FGDs followed semi-structured guides and focused on domains that included experiences of sexual and physical violence; sexual behavior (sexual debut, practices, and partners); experiences disclosing sexual orientation; and mental health. IDIs and FGDs were conducted in Mongolian, professionally transcribed, and translated into English by bilingual native Mongolian speakers.

Among the full set of interviews and FGDs, sexual violence was explicitly discussed in 28 IDIs and 2 FGDs (semi-structured guides allowed content of each interview to vary). We defined sexual violence in the qualitative phase according to Centers for Disease Control and Prevention guidelines, including nonconsensual completed or attempted sexual penetration or contact, and nonconsensual non-contact sexual acts such as voyeurism and verbal or behavioral sexual harassment [32]. Childhood sexual abuse and sex as a minor (legally unable to consent) with an older individual are included in this definition. This broad definition was intended to capture the full range of nonconsensual sexual experiences over the life course of each respondent and fit well with the exploratory nature of the typology analysis, described below. Interview transcripts were read in totality by one researcher. Text that described a sexually violent incident, denied an incident was sexually violent, or discussed general attitudes toward consent, sexual coercion, or sexual violence were topically coded for further analysis.

Data were managed using ATLAS.ti (Version 7.0.71; ATLAS.ti GmbH; Berlin, Germany). As borrowed from a grounded theory approach [33], initial line-by-line open coding was conducted by the first author, followed by focused and axial coding. Constant comparative analysis was used to link codes and develop themes across and within texts. When additional context was needed during analysis, the original full transcripts were referenced and additional text was coded. Memos were used to organize ideas, and key themes were discussed among the research team.

Faced with a large number of heterogeneous violent incidents in the data, constructing a typology of sexual violence against MSM and transgender women emerged as a key aim of the qualitative analysis. Rape typologies arose in the 1970s and 1980s in the criminal justice and psychology literatures [34, 35]. These typologies “taxonomically” classify the behavioral, motivational, and cognitive characteristics of perpetrators [36, 37], and have been used to better understand the etiology of rape, as well as to predict perpetrator behavior, assess risk of recidivism, and identify appropriate treatment for offenders based on type [37, 38]. Data in the present study are largely collected from victims and bystanders, so our typology is not perpetrator-focused. Rather, we construct an incident-focused typology that describes the most common types of sexually violent experiences by exploring characteristics of the location where the incident occurred, the relationship between the victim and perpetrator, and the means used to perpetrate the violence. A similar exploratory typology has been constructed to describe the issue of wartime rape so that it can be better understood, recognized, and addressed in the field [39]. Such classifications are useful in better understanding the heterogeneous experiences that comprise the phenomenon of sexual violence in a population and can be used to design survey tools that more clearly and comprehensively assess the prevalence of sexual violence in its many forms. It can also be used to design interventions tailored for specific locations, types of perpetrators, or means of perpetration. An empirical description of common types of sexual violence may also be helpful in improving resource allocation to address types of violence that were previously under-recognized [39] and increasing the willingness of jurors and others to recognize a non-stereotypical event as sexually violent [40].

Once a typology has been constructed, understanding vulnerabilities to different types of sexual violence can help identify individuals who are at greatest risk of victimization and therefore who would most benefit from prevention interventions. Finally, understanding sequelae to different types of sexual violence can suggest important secondary prevention interventions and victim support services. Following the typology, we therefore present themes that emerged as vulnerabilities to sexual violence and sequelae of sexual violence. Where data permit, we link specific vulnerabilities and sequelae to the types of violence identified by the typology for which they are particularly relevant.

### Ethics

This study was approved by the Medical Ethics Board of the Mongolian Health Science University’s School of Public Health and the Johns Hopkins Bloomberg School of Public Health Institutional Review Board. All participants provided written informed consent, with the option of using a mark or giving oral consent in lieu of signing their name for privacy, before questionnaires were implemented. The legal age for provision of consent in Mongolia is 16; therefore, inclusion of minors aged 16 and 17 without parental consent was approved by the ethics boards.

## Results

### Prevalence and Correlates of Sexual Violence

Participants were a median age of 28 years and 89% identified as male-gendered (Table 1). Some 14.7% (CI: 9.4–20.1) reported experiencing forced or unwanted sex in the past three years. There were no statistically significant differences (p = 0.467) in the prevalence of forced sex between transgender (6/29, 20.7% crude percentage) and male-identified respondents (43/278, 15.6% crude percentage), but the power to detect these differences was limited due to the small number of transgender individuals (Table 2). Other physical and social violence included lifetime experiences of verbal harassment (54.8%), physical or verbal abuse by police (8.5%), and physical violence (10.4%). Some 16.2% of participants (95% CI: 9.3–24.4) reported a history of incarceration.

**Table 1:** Forced Sex and Estimates of Selected Risk Factors among MSM in Ulaanbaatar, Mongolia.

Risk factor	Crude Total % (n/N)	RDSAT-weighted % (95% Confidence Interval)
Demographics		
Age, mean/median, interquartile range (range)	30.8/28.0, 22–40 (16–62)	
Tertiary or greater education	57.5 (180/313)	46.8 (38.2–55.6)
Greater than 250k MNT/month income	45.0 (140/311)	37.6 (28.9–45.8)
Transgender	9.3 (29/311)	10.8 (5.2–17.4)
Depressive symptoms	59.7 (187/313)	56.2 (47.6–66.5)
Ever had STI diagnosis	12.1 (28/232)	11.6 (4.5–19.6)
Ever had HIV diagnosis	7.3 (16/219)	6.3 (1.1–12.1)
Sexual Behavior		
Always receptive partner	10.6 (33/312)	12.6 (6.7–18.5)
Ever received payment for sex^a^	10.5 (33/313)	5.4 (2.7–8.2)
Drink alcohol at least once a week	39.3 (123/313)	29.7 (21.2–36.6)
Ever unable to remember night before because of drinking (past 12 months)	60.3 (179/297)	60.6 (50.7–68.9)
Ever have alcohol before or during sex	74.4 (232/312)	64.4 (55.1–72.4)
Greater than 5 male casual partners (past 12 months)	24.9 (78/313)	13.7 (9.1–18.3)
First anal sex encounter		
Boyfriend	19.0 (59/310)	25.2 (16.1–33.8)
Friend	21.0 (65/310)	17.9 (12.6–23.7)
Relative	2.3 (7/310)	3.0 (0.3–6.9)
Someone else they knew	31.9 (99/310)	35.1 (27.0–45.1)
Stranger	14.5 (45/310)	12.2 (7.1–19.0)
Social, Physical, and Sexual Violence (past 3 years unless otherwise indicated)		
**Forced Sex**	**16.0 (49/307)**	**14.7 (9.4–20.1)**
Felt that police refused protection or ignored you^b^	9.8 (30/307)	6.3 (3.2–9.9)
Verbal or physical abuse by police^b^	14.4 (45/312)	8.5 (4.8–12.3)
Blackmailed by police^b^	9.6 (30/313)	7.2 (3.6–10.7)
Verbally or psychologically harassed^b^	55.8 (169/303)	54.8 (46.4–63.7)
Felt legal discrimination^b^	34.0 (102/300)	34.8 (27.5–45.6)
Physically harassed or beaten up^b^	13.7 (43/313)	10.4 (6.0–15.1)
Felt excluded from family gatherings^b^	4.5 (14/312)	3.5 (1.5–6.1)
Felt rejected by friends and other MSM^b^	32.4 (101/312)	27.6 (20.6–34.8)
Ever been estranged from family^b^	7.1 (22/312)	4.0 (2.0–6.8)
Ever disclosed MSM status to family member	21.1 (66/313)	16.5 (9.4–22.4)
Ever disclosed MSM status to healthcare worker	46.2 (144/312)	28.7 (20.4–35.7)
Ever been to jail	15.3 (48/313)	16.2 (9.3–24.4)
Ever been to detox/drunk tank	38.0 (119/313)	33.3 (22.5–42.6)

^a^ payment includes money, drugs, food, shelter and transportation.

^b^ “as a result of sexual orientation or gender identity or practice.”

**Table 2:** Correlates of Forced Sex among MSM in Ulaanbaatar, Mongolia. Results of bivariate and multivariate logistic regression analyses (using backward selection).

Risk factor	Forced Sex in past three years		OR	AOR
	Yes, n (%)	No, n (%)	(95% Confidence Interval; p)	(95% Confidence Interval; p)
Demographics				
Age (continuous)	-	-	0.99 (0.96, 1.02; p = 0.375)	-
Tertiary or greater education	29 (16.38)	148 (83.62)	1.08 (0.58, 2.01; p = 0.813)	-
Greater than 250k MNT/month income	23 (16.67)	115 (83.33)	1.08 (0.58, 2.00; p = 0.795)	-
Transgender	6 (20.69)	23 (79.31)	1.41 (0.54, 3.68; p = 0.478)	-
Depressive symptoms	29 (15.68)	156 (84.32)	0.95 (0.52, 1.77; p = 0.867)	-
Ever had STI diagnosis	5 (17.86)	23 (82.14)	1.22 (0.43, 3.47; p = 0.703)	-
Ever had HIV diagnosis	1 (6.25)	15 (93.75)	0.34 (0.04, 2.70; p = 0.309)	-
Sexual Behavior				
**Always receptive anal sex partner**	**10 (31.25)**	**22 (68.75)**	**2.74 (1.21, 6.22; p = 0.016)**	**3.65 (1.46, 9.13; p = 0.006)**
**Ever received payment for sex** ^**a**^	**12 (37.50)**	**20 (62.50)**	**3.86 (1.74, 8.55; p = 0.001)**	**2.77 (1.14, 6.75; p = 0.025)**
**Drink alcohol at least once a week**	**31 (26.05)**	**88 (73.95)**	**3.33 (1.76, 6.28; p<0.001)**	**3.39 (1.69, 6.81; p = 0.001)**
Ever unable to remember the previous night because of drinking (past 12 months)	34 (19.32)	142 (80.68)	1.73 (0.88, 3.38; p = 0.111)	-
**Ever have alcohol before or during sex**	**42 (18.50)**	**185 (81.50)**	**2.33 (1.00, 5.44; p = 0.049)**	-
Greater than 5 male casual partners (past 12 months)	14 (18.18)	63 (81.82)	1.24 (0.63, 2.45; p = 0.539)	-
First anal sex encounter				
Boyfriend	5 (8.47)	54 (91.53)	1.00	-
Friend	12 (18.46)	53 (81.54)	2.45 (0.81, 7.41; p = 0.114)	-
Relative	2 (28.57)	5 (71.43)	4.32 (0.66, 28.27; p = 0.127)	-
Someone else they knew	15 (15.62)	81 (84.38)	2.00 (0.69, 5.83; p = 0.204)	-
**Stranger**	**12 (26.67)**	**33 (73.33)**	**3.93 (1.27, 12.14; p = 0.018)**	-
Social, Physical, and Sexual Violence (past 3 years unless otherwise indicated)				
Felt that police refused protection or ignored you^b^	8 (26.67)	22 (73.33)	1.01 (0.99, 1.03; p = 0.156)	-
Verbal or physical abuse by police^b^	14 (31.11)	31 (68.89)	0.99 (0.91, 1.07; p = 0.811)	-
Blackmailed by police^b^	8 (26.67)	22 (73.33)	0.98 (0.88, 1.10; p = 0.757)	-
Verbally or psychologically harassed ^b^ ^,^	32 (19.16)	135 (80.84)	1.83 (0.95, 3.55; p = 0.073)	-
Felt legal discrimination^b^	15 (14.71)	87 (85.29)	0.93 (0.48, 1.82; p = 0.835)	-
Physically harassed or beaten up^b^ ^,^	8 (18.60)	35 (81.40)	1.24 (0.54, 2.87; p = 0.610)	-
**Felt excluded from family gatherings** ^**b**^	**6 (42.86)**	**8 (57.14)**	**4.55 (1.50, 13.81; p = 0.007)**	-
**Felt rejected by friends and other MSM** ^**b**^	**22 (22.00)**	**78 (78.00)**	**2.12 (1.12, 3.99; p = 0.021)**	1.83 (0.93, 3.61; p = 0.083)
**Ever been estranged from family** ^**b**^	**7 (31.82)**	**15 (68.18)**	**2.69 (1.03, 6.99; p = 0.042)**	-
Ever disclosed MSM status to family member	9 (13.85)	56 (86.15)	0.81 (0.37, 1.77; p-0.601)	-
Ever disclosed MSM status to healthcare worker	26 (18.57)	114 (81.43)	1.42 (0.77, 2.62; p = 0.264)	-
Ever been to jail	6 (12.77)	41 (87.23)	0.74 (0.30, 1.85; p = 0.517)	-
Ever been to detox/drunk tank	17 (14.55)	99 (85.34)	0.85 (0.45, 1.62; p = 0.627)	-

^a^ payment includes money, drugs, food, shelter and transportation.

^b^ “as a result of sexual orientation or gender identity or practice.”

In the multivariate model, always being the receptive partner (Adjusted Odds Ratio (AOR): 3.65; 95%CI: 1.46–9.13), weekly alcohol consumption (AOR: 3.39), and lifetime history of receiving payment for sex (AOR: 2.77; 95%CI: 1.69–6.81) were independently associated with sexual violence in the last three years (Table 2). Feelings of rejection by friends and other MSM was marginally associated with sexual violence (AOR: 1.83; 95%CI: 0.93–3.61). Alcohol use during or prior to sex, first sex with a stranger, and several social exclusion variables were significant under bivariate analysis but were no longer significant in the multivariate model.

### Qualitative Typology

Out of the 28 interviews and 2 focus groups where sexual violence was mentioned, a specific sexually violent incident was discussed in 15 interviews and one focus group. Thirty-seven incidents of sexual violence or coercion were described. The study respondent was the victim in 20 of these incidents, an eyewitness in five, heard directly from the victim in seven, and heard of the event indirectly (secondhand or media reports) in five. Thirty-one incidents were described by MSM (14 respondents) and six incidents were described by transgender women (two respondents). Participants ranged in age from 18 to 58 (median age 28 years).

Eight major types of sexual violence were identified (Table 3). Sexual violence types were either closely tied to risky locations (street violence, party-related violence, and incarceration-related violence), which we term venue-specific, or were based on relationship power dynamics and violence tactics and occurred in a variety of settings (police-perpetrated violence, intimate partner violence, child sexual abuse, or sexual violence achieved through coercion or to avoid disclosure of sexual orientation), which we term relationship- and tactics-specific.

**Table 3:** Descriptive typology of sexual violence against MSM. Qualitative matrix of violence type, location, tactics and analysis of perpetrator-victim relationship among study participants in Ulaanbaatar, Mongolia, 2011.

Violence Type	Location	Violence Tactics	Perpetrator(s) and Relationship to Victim
**Location-Specific sexual violence**			
**Street violence**	Street, or abducted in car on street	Physical force and/or abduction, threat of physical force, and verbal abuse.	Usually non-gay-identified male strangers; female perpetrators of verbal abuse reported. Multiple perpetrators.
**Party-related violence**	House party	Alcohol incapacitation (intentional or opportunistic)	Gay-identified, new acquaintances. Single or multiple perpetrators.
**Incarceration-related violence**	Jail	Physical force	Other inmates, often those who have been there longer. Unclear sexual identities. Often multiple, sometimes single perpetrator.
**Relationship or Violence Tactics-specific sexual violence**			
**Coercion through blackmail or to avoid disclosure**	Anywhere	Victim coerced into sexual activity through blackmail, or to “prove” he is not MSM	Various (gay-identified men, non-gay-identified men, women). Single perpetrator.
**Commercial sex-related violence**	Hotel	Client expectations of consent during commercial sex, verbal pressure, physical force	Client, gay-identified. Single or multiple perpetrator(s).
**Police-perpetrated violence**	Police station, street	Institutional power, physical force	Non-gay-identified male police officers. Unknown to victim. Usually multiple perpetrators.
**Child sexual abuse**	Home of victim, home of perpetrator, other	Power through age disparity and youth of the victim	Older person known to and trusted by the victim, e.g. neighbor, older friend, or relative. Single or multiple perpetrators; male or female.
**Intimate partner violence**	Shared home or public area	Taking advantage of trust or economic need to remain in relationship.	Current or former sexual partner with whom sex was at some point consensual. Single perpetrator.

#### Scenario 1: Street Violence

Sexual violence and harassment from strangers in public places, motivated by hostile attitudes toward the victim’s sexual orientation and gender identity, was the most commonly reported type of sexual violence. Derogatory terms referring to gay men and transgender women were common.

They say you damn *bandi*, you damn *shuumar*, you wanna get fucked? Even they say, why not just rape you and slay you…When [friends] were traveling on a train, a guy told them “Are you all *bandi*, nobody can tell if you’re male or female, I’ll push a dagger into your shitter.”…Such things happen a lot (p21, MSM)

One participant described his experience of abduction and gang rape:

It was at the party in February…I went outside to get a phone call and three guys forcefully put me in the car…There they took my cell phone and jacket; they left me in my pants and shirt in the cold winter night…Besides me, two other guys were captured that night. They put me in the car and [raped me]…I heard they were talking over the phone, “We have someone here. The others have another one. Did you find someone?” (p23, MSM).

#### Scenario 2: Party-Related Violence

Party-related sexual violence was the second most commonly reported scenario. Perpetrators would intentionally provide the victim with alcohol or wait until an individual was too inebriated to resist unwanted sexual advances. One respondent perceived party-related sexual violence as a typical part of initiation into the community:

I joined the gay community…I was 20…On that day I got really drunk out of my mind. When I woke up the next morning, I had this terrible pain, and there were five naked people lying next to me. I did not understand anything that had happened. Anyway, by then, I realized what they did with people coming out recently or anew…There is violation. Whenever there is a new person, former people pass him to each other as if he is a ball (p29, transgender).

Another respondent described a situation in which one might have consensual sexual activity, but pass out afterward and be violated by someone else.

People drink together when there are a whole bunch of them, right? So when there are two people who are attracted to each other, they drink vodka and other alcoholic beverages. Then they black out and when one of them wakes up early in the morning, he finds himself together with a totally different person. (p14, focus group MSM)

#### Scenario 3: Violence during Incarceration

Respondents reported forced anal penetration by one or multiple fellow inmates while in prison. One respondent had heard from a victim that rape was widespread in prisons; this victim was “first imprisoned when he was 17 years old…There were seven other prisoners other than him and all those prison bosses pulled him down and raped him… according to him, those people would always rape every single young and new prisoner” (p14, focus group MSM).

#### Scenario 4: Coercion through Blackmail or to Avoid Disclosure of Sexual Orientation

This category includes heterogeneous experiences, with the only commonality being that victims were coerced into sex to avoid being ‘outed’ (nonconsensual disclosure of their sexual practices to others).

One participant described a situation in a rural area of Mongolia when older MSM attempted to pressure an adolescent into sex, threatening “I will tell your family members about you” (p3, MSM). Sexual violence was sometimes perpetrated by women as well. One respondent reported having been coerced into sex separately by two female friends, one of whom said to him, “If you are not a gay, have sex with me. Prove yourself” (p8, MSM). To avoid disclosing his orientation, he felt he had to comply.

#### Scenario 5: Violence Related to Commercial Sex

Two respondents reported sexual violence related to commercial sex, facilitated by client-sex worker power dynamics. Once money had been exchanged, clients expected the right to demand any type of sexual act. One respondent described his experience when a client brought another man to the sexual engagement, demanding the sex worker provide sex for both men.

The other one says to me, “He has paid all hotel payments. Stop being ridiculous! You have had many sexes before. I actually heard of you from people. Stop acting like an 18 year old virgin!”…They say like that and take off my underwear. That’s how I am almost forced to have sex with them. (p3, MSM)

#### Scenario 6: Police-Perpetrated Violence

Some respondents reported violence from police, including one transgender woman who described being assaulted when walking in the street with her friends. Men and women threw stones at them, spilled urine on their faces, and verbally harassed them for “offending the reputation of men.” When they complained to the police, the police responded with:

“Who the hell asked you to dress as girls and wander around?” they say. They were swinging their blackjacks, lifted up our skirts, saying, “How do you have sex with each other? Do it now before us, suck the things for one another…You do it because you like it, you ugly hustlers, you offend man’s pride, now have sex with each other, you suck mine…Lift your skirt, or d’you wanna suck mine, I’ll push the blackjack into your ass.” (p31, transgender)

#### Scenario 7: Child Sexual Abuse

Three respondents reported sexual violence during childhood or adolescence. One respondent recalled an instance in which a man paid a family to lock their son in his house for sex. Another participant stated that when he was 15, three friends of his father’s came to stay overnight in his home while his family was away. After drinking vodka, the three men sexually assaulted him, and the “next morning, they told me that they would kill me if I told my parents” (p 29, transgender). This participant also stated, “It seems like such a thing happens a lot, in particular, to teenagers who are feminine.” This individual also attributed the incident in large part to the influence of alcohol, saying, “Oh well, you know that people’s attention is diverted to sex when they drink.” Another respondent reported having been abused by an older adolescent female when he was about 8 years old.

#### Scenario 8: Intimate Partner Violence

Intimate partner violence was described by three respondents. Reported IPV included forced sex while the perpetrator was intoxicated, date rape, and intentionally causing pain during sexual intercourse as a form of punishment. Physical abuse was also reported.

#### Miscellaneous Sexual Violence

A few incidents did not fit neatly into any category, and further research is needed to understand if they warrant a separate category in the typology. A potential category of *sexual contact with a minor* was identified: Two secondhand reports of sex with a minor were mentioned in which a teenager under the legal age of consent (under 16) was in a long-term sexual relationship with someone who had achieved the age of majority. The respondents did not identify the minors as having been coerced into the relationship, but the minors were legally unable to consent to sexual activity. A second potential category of *military-related sexual violence* was identified: Two additional participants reported being subjected to unwanted sexual touching by their ranking officers.

### Vulnerabilities to Sexual Violence

Four factors that contributed to sexual violence vulnerability across multiple scenarios were 1) social stigma and discrimination, 2) alcohol use by victim or perpetrator, 3) economic vulnerability, and 4) youth.

#### Social Stigma and Discrimination

Stigma against homosexuality increased vulnerability to every type of sexual violence. Police-perpetrated violence and street violence were motivated by homophobic/transphobic attitudes. Because respondents often faced harassment when congregating in public bars, there was a reliance on house parties hosted by a member of the MSM/transgender community to socialize and meet sexual partners; unfortunately, not all respondents were safe from sexual violence in these settings.

Social stigma particularly enabled sexual violence through coercion or blackmail, often to avoid disclosure of sexual orientation or practices. Only 17% of participants in the quantitative study reported disclosure of their identity to any family member and 29% reported disclosure to health care workers, suggesting that the majority of MSM and transgender women in Mongolia do not voluntarily disclose their sexual orientation or practices.

One qualitative respondent stated that his friend was arrested on false charges, potentially due to legal discrimination against MSM, and placed in jail, where he was gang raped.

#### Alcohol Use

Alcohol use by the victim or perpetrator was explicitly mentioned or strongly implied in 15 of the sexual violence incidents described by respondents. Heavy alcohol use was particularly common for party-related violence. Because these parties are a primary means of finding sexual partners, this sometimes led to an expectation of sex; i.e. that “some of them used to have this intention of having sex with this and that person no matter what happens” (p11, MSM). Alcohol use by the perpetrator was also mentioned in cases of IPV, street violence, child sexual abuse, and violence related to commercial sex.

#### Economic Vulnerability

Economic vulnerability enhanced risk of several types of sexual violence, including commercial sex-related violence, street-related violence, party-related violence, and intimate partner violence. Transgender respondents in particular reported many of their peers “cannot work because of discrimination,” leading them to begin selling sex and exposing them to violence from passers-by and clients (p31, transgender). Respondents reported going to a party or to meet someone and not having sufficient money for transportation home, leading them to spend the night with the person and sometimes be subject to unwanted sexual advances. Another man who reported experiencing intimate partner violence was forced to stay with his partner due to lack of legal documentation and being forced to rely on his partner to provide housing and assistance with employment.

#### Youth

Young age was a cross cutting vulnerability in many types of sexual violence, including violence that occurred in the context of commercial sex, party-related violence, blackmail or to avoid disclosure, and child abuse. At least 17 of the reported sexual violence incidents featured a young or sexually inexperienced victim. Youth often overlapped with economic and alcohol related vulnerabilities. Many respondents indicated that younger MSM were more prone to engaging in commercial sex because they had limited funds and were thus vulnerable to older MSM who had the disposable income to pay for sex. The following quote demonstrates that many young men may be more vulnerable to violence:

This happens to those who are just coming out, those young boys who are just coming out to the community. Because they are defenseless, because they are unable to resist, they are most susceptible. It happened to me many times when I didn’t want it…the excessive drinking also affects it. Boys who are just coming out are forced to drink a lot. And I went through exactly that way. I was just sitting and drinking and there was a gentleman of forty or fifty years old. I fell down unconscious…And when I wake up and see that gentleman is already attempting to insert it. And it was horrible that he was not using a condom…I tell him not to do it, that he shouldn’t, but I’m not as strong as him. So I had to admit it silently, and in the morning I got up powerless, I had very awkward feelings. It was horrible…Because it was the time of my just coming out, I felt a terrible pain, and looking at my clothes, I understood it has happened and, I got horribly embarrassed as if I, myself, raped someone, feeling guilty…it was just like a nightmare. (p31, transgender)

### Secondary Victimization and Health-Related Sequelae following Sexual Violence

After disclosing an incident of sexual violence to the authorities, peers, or family members, respondents reported experiencing or expecting secondary victimization, including physical, social, sexual, and emotional violence. Sexual and mental health sequelae were also reported.

#### Abuse from Police

One respondent said that after being raped, there was “no use reporting to the police. The police would say, ‘You deserve it. People like you do not have rights’” (p23, MSM). In an interaction with police after being accused of a crime, they had told him “you are gay. Human rights do not apply to people like you. You are not a human being.” Other participants reported physical abuse from police.

…There is no protection for our community. If we were victims, we will stay victims…If we become victims of street beating and go to the police station, we will become double victims after being beaten up by police. They do not rape, I do not know about that for sure. [But] there are lots of problems like beating, slandering, jailing without any reason, and torturing…They cuffed me to the heater in the criminal detective’s room, left me like this for three days, and knocked me off with the baton to unconsciousness. (p23, MSM)

#### Lack of Social Support

MSM and transgender women reported feeling blamed by others for “tempting” the perpetrator or behaving in a feminine manner. Due to these social perceptions, many victims reported feeling unable to seek support from their family and peers for fear of being blamed. One respondent stated, “If I tell this to my gay friends, they’ll not believe that I didn’t want it…I’ve never told anyone about it” (p31, transgender). Many respondents had concealed their sexual or gender identity from their family and felt they could not seek support for their victimization without revealing their identity: “I did not tell [my family]. How [laughs] can I tell? They would be surprised” (p23, MSM).

#### Need to Conceal Sexual Identity

Social stigma also influenced access to justice for survivors of sexual violence. A respondent explained that reporting sexual violence to authorities was difficult due to concerns that the victim’s sexual orientation would be revealed as part of the court proceedings, and that male survivors of sexual violence were not protected equally under law:

What will you gain by filing a case, where can you complain to, the cops? The charged will also say, “This boy is a gay, too.” Then the family will be called…Their human rights are violated…of course the young person can’t appeal to the cops or court, and the case remains hidden. Okay, let’s assume that the family knows that their son is gay anyway. When they come to court, it’ll be funny, cause it’s a rape of a man by a man, they change the case into satisfaction of one’s sexual desire with an inappropriate method. This gives the charged just a few months’ sentence. Cause the victim isn’t a woman, he’s a man. (p31, transgender)

#### Persecution of the MSM and Transgender Community

When the rape of a community member is reported, the MSM/transgender community may be persecuted as a result, even when the perpetrator(s) identifies as heterosexual and committed the act as a hate crime:

About seven or eight years ago, a guy called L was raped to death by four people. Four straight people committed this crime while drunken. But then, they interrogated all of us [MSM and transgender women], making us stand in a line that stretched from the first floor to the second one. (p29, transgender)

Media coverage of sexual assault against MSM and transgender women also victimized the LGBT community. The media characterized this community as sexually predatory, particularly toward children. One respondent observed,

From the beginning, [two well-known transgender women] were reported on the media and press that they lured children under age. People became disgusted because of that. It gave the people an ugly perception that these homo people seduce young children (p28, MSM).

#### Rape-Related HIV/STI Risk

When sexual violence occurred in the context of heavy alcohol use, respondents often did not even know who had raped them, exposing them to unknown HIV risk. Victims reported being informed at a later date who raped them by friends who had casually witnessed the event:

When we are together someone says to the other “Hey, you are infected. [Perpetrator] was riding on you. Go and get tested, bugger.” The other person then replies, “How could I know? I was totally passed out. He was taking advantage of me…” (p8, MSM).

Several survivors specifically mentioned that condoms and lubricant were not used during sexual victimization. One participant described, “He practically took me away by force and did it. He…pushed me to the wall next to a rubbish pipe and did it without a condom, without a lubricant, he just used his saliva.” (p21, MSM) Another victim was raped in jail and later found he was HIV-positive, which he attributed to that experience.

#### Mental Health

The anxiety of fearing violence was described thusly: “When we’re getting ready to walk out, we make up, dress up, very happy. Then while already in the street, we are worrying, what will happen now, what will they do to us. Stoning is a simple thing, even an eight year old can do it. This psychological pressure, stress may be even worse than physical pain” (p31, transgender).

Some participants associated sexual violence with feelings of confusion over sexual identity or feelings of being “turned” gay or transgender, particularly if the sexually violent incident was the victim’s first sexual contact with another man. These feelings may contribute to the mental health burden of a population experiencing high prevalence of depressive symptoms.

## Discussion

Taken together, these data suggest that sexual violence commonly affects MSM and transgender women in Mongolia. The prevalence of sexual violence reported in this study is consistent with studies from other low- and middle-income settings [16, 25]. In the qualitative data, the vulnerability associated with alcohol use and youth and the riskiness of parties and social venues echoed themes from existing research in high-income settings [14, 19, 41], but also affirmed the common presence of violence on the street and in other scenarios. There was no universal experience of sexual violence: Sexually violent incidents were heterogeneous with respect to location, coercion tactics, vulnerabilities, and relationship between the victim and perpetrator. This diversity indicates that a variety of structural, venue-based, and behavioral interventions should be developed and tailored to target specific vulnerabilities and types of violence.

Results from quantitative and qualitative data were consistent, and together offer a description of the types of violence experienced as well as some understanding of their impact at a population level. Several factors identified by qualitative participants as vulnerabilities to sexual violence (such as alcohol use) and consequences of sexual violence (such as social isolation) were also significantly associated with recent forced sex in the bivariate or multivariate analyses. Selling sex was associated with forced sex in the quantitative data, while in the qualitative data, commercial-sex related violence was identified as a major type of violence in the typology and economic vulnerability was identified as a vulnerability to sexual violence as well as an impetus to sell sex. Quantitative results also offer some insight into what proportion of the population may be at risk of different types of sexual violence. For instance, 16.2% of quantitative participants reported a history of incarceration and were at risk of incarceration-related violence, while 5.4% reported ever selling sex, placing them at risk of commercial sex related violence. Only 16.5% of quantitative participants reported disclosure of their MSM status to any family member, suggesting that the majority of MSM and transgender women in Mongolia may be vulnerable to coercion to avoid disclosure of their sexual orientation or gender identity. The fact that party-related violence was one of the most commonly mentioned forms of violence in the qualitative data was concordant with the high past-year prevalence of drinking to the point of blackout (60.6%) and the association of alcohol use and forced sex in the quantitative data.

Evidence-based violence prevention programs have evolved from promoting individual risk reduction to identifying and addressing structural drivers of violence at a population level. The data presented here suggest that structural interventions may be particularly important at reducing sexual violence against MSM and transgender women in Mongolia. Limited law enforcement protection for this population facilitated a culture of impunity among perpetrators of street violence, with police sometimes engaging in abuse themselves. In addition to facilitating street violence, lack of protection in public spaces also drove the popularity of more secluded house parties where MSM and transgender women could associate without harassment. Unfortunately, house parties were sometimes reported as risky venues for socializing, due to the control of alcohol by older individuals and the presence of private bedrooms where assault can occur. This parallels research from college campuses that has demonstrated that structural and venue-level factors foster a rape-enabling environment. For example, strict alcohol enforcement policies in freshman dormitories concentrate alcohol in the hands of unpoliced, off-campus fraternity houses controlled by older male students who are then able to control women partygoers’ “transportation, admission, access to alcohol, and movement” [42]. Improving police response to public harassment of MSM and transgender women would not only deter street violence and police violence, but could also reduce party-related violence by providing safe public spaces for MSM and transgender women. The Mongolian law concerning rape addresses only rape of female persons [6], limiting access to care and preventing prosecution for sexual violence against males. This lack of legal recognition increases opportunities for victim blaming and stigmatization [43], resulting in negative mental health outcomes, and drives impunity for perpetrators, facilitating further abuse.

Successful structural interventions to prevent violence against other key populations may be adaptable to MSM and transgender women in Mongolia. For female sex workers, integrated, structural approaches to preventing violence that combined advocacy with policy makers, trainings with police officers, education and mobilization of sex workers, and creation of peer-led crisis-response teams were effective in significantly reducing violence against sex workers in Karnataka state, India [44]. This approach could be adapted to MSM and transgender populations facing street violence, violence related to commercial sex, and violence from police.

The two most common types of sexual violence reported, street-related violence and party-related violence, were both location-specific, suggesting venue-based interventions to reduce sexual violence risk should be investigated. Violence prevention has moved toward venue-based interventions that make perpetrating violence appear “more risky, more difficult, less rewarding, and less excusable” and increase the ability of the potential victim to detect and ward off threats [45]. Similarly, there has been a movement in HIV prevention toward venue-based interventions that target high-risk people where they engage in high-risk behaviors, such as venues that sell alcohol or where sex is sold [46, 47]. Venue-based sexual violence-prevention interventions could be integrated into venue-based HIV prevention programs due to the overlap between venues with sexual risk and risk of sexual violence, particularly sexual violence related to alcohol consumption, parties, and sex work. For instance, in the US, attendance at circuit parties (multiday parties attended by thousands of MSM) is associated with increased drug use and sexual risk behavior. Given the venue-specific concentration of risk, location-specific prevention messaging, condom distribution, bathroom attendants, adequate bathroom lighting, and other venue-specific efforts can reduce risk for both HIV and violence [48, 49]. Venue-based risk in public places such as bars and in the street could potentially be addressed through venue-based interventions such as improved police monitoring of the areas (pending improved police training) and better lighting. Although instituted in a different population and setting, these approaches have been successful to improve venue-based sexual violence risk for women in refugee camps who are assaulted in public spaces [50, 51].

Behaviorally based rape-prevention programs could also be adapted for sexual violence prevention in the Mongolian MSM and transgender community. Party-related sexual violence often occurred in plain sight of other partygoers. Bystander intervention programs that address interpersonal factors in sexual violence prevention and have been successful in decreasing acceptance of rape myths, increasing the number of bystander intervention behaviors, reducing likelihood of rape perpetration, and increasing empathy for victims following an assault in other populations [52, 53]. Alcohol use was identified as a risk factor for sexual violence in both quantitative and qualitative phases and could be a good target for behavioral intervention. While alcohol does not directly cause sexual assault, it can make victims less able to recognize and resist assault, and may provide perpetrators with an excuse for their behavior [54]. The majority of quantitative participants had at least one night in the last year they could not remember because of inebriation and reported alcohol use before or during sex, suggesting alcohol-related vulnerabilities may be important to address in population-level sexual violence prevention. As Fenaughty and colleagues note, “In drawing attention to the issue of sexual coercion among gay and bisexual men there is a risk of other people utilizing this information in ways that pathologize, and result in increased discrimination against, an already marginalized community” [41]. Behavioral interventions should be a component of a comprehensive intervention package that also targets structural and venue-based risk.

Secondary prevention programs designed to minimize harm following a sexually violent incident should also be enhanced. The majority of participants reported depressive symptoms and survivor testimony from the qualitative phase suggest that survivors’ physical and mental health needs are not being met. Services such as crisis intervention counseling, ongoing therapy, medical referrals, forensic exams, and post-exposure STI and HIV prophylaxis should be made available. Providers should ensure confidentiality and privacy, pay particular attention to sexual violence that MSM and transgender women may disproportionately experience such as hate crimes, address feelings of internalized homophobia or transphobia following sexual assault, provide literature with male-specific or gender-neutral language, and conduct forensic exams designed for male bodies as appropriate [55].

More research on sexual violence in this population is warranted. Importantly, more structural, venue-based, and behavioral interventions specifically tailored to MSM and transgender communities in diverse settings should be developed and evaluated, as rape prevention literature has been dominated by interventions conducted among college students in high-income countries with a focus on violence against women [56]. Collecting additional accounts of sexual violence from Mongolian MSM and transgender women will strengthen the proposed typology and identify significant types of sexual violence that may be missed. This typology should be validated and refined in broader samples to ensure generalizability among Mongolian MSM and transgender women, and to understand its transferability to other settings. Quantitative data should be collected to understand the incidence of the specific types of violence identified in our typology and their potentially different effects on health. Transgender respondents should be more heavily recruited in future work to understand how sexual violence might differ by gender identity. This study was not powered on experiences of sexual violence, thus we lacked the sample size to detect some associations that may exist. The question used to assess sexual violence in the quantitative study may have underestimated sexual violence as it only asked about forced sex, and may not have captured attempted sexual violence, acts of coercion, and non-penetrative sexual violence. Limitations to this study also include its cross-sectional nature, prohibiting causal, temporal inference.

Understanding where, how, and how often violence occurs is the first step in addressing sexual violence in a population. These data should inform the development and implementation of comprehensive interventions in partnership with MSM and transgender communities, as well as with the government, law enforcement, and broader Mongolian society, to prevent sexual violence and improve sexual and mental health among these men and women.
